# Brain activation during an emotional task in participants with PTSD and borderline and/or cluster C personality disorders

**DOI:** 10.1016/j.nicl.2023.103554

**Published:** 2023-12-18

**Authors:** Inga Aarts, Chris Vriend, Odile A. van den Heuvel, Kathleen Thomaes

**Affiliations:** a Sinai Centrum, Arkin, Amstelveen, the Netherlands; b Amsterdam UMC, Vrije Universiteit Amsterdam, Department of Psychiatry, Department of Anatomy and Neurosciences, Boelelaan 1117, Amsterdam, the Netherlands; c Amsterdam Neuroscience, Compulsivity, Impulsivity & Attention program, Amsterdam, the Netherlands

**Keywords:** PSTD, Borderline Personality Disorder, Cluster c personality disorder, Emotional face task, Avoidant personality disorder, fMRI

## Abstract

**Introduction:**

Although comorbidity of post-traumatic stress disorder (PTSD) with borderline personality disorder (BPD) and/or cluster C personality disorders (CPD) is common, neural correlates of this comorbidity are unknown.

**Methods:**

We acquired functional MRI scans during an emotional face task in participants with PTSD + CPD (n = 34), PTSD + BPD (n = 24), PTSD + BPD + CPD (n = 18) and controls (n = 30). We used ANCOVAs and Bayesian analyses on specific ROIs in a fearful vs. scrambled faces contrast. We also investigated associations with clinical measures.

**Results:**

There were no robust differences in brain activation between the groups with ANCOVAs. Transdiagnostically, we found a negative association between severity of dissociation and right insula and right dmPFC activation, and emotion regulation problems with right dmPFC activation. Bayesian analyses showed credible evidence for higher activation in all ROIs in the PTSD + BPD + CPD group compared to PTSD + BPD and PTSD + CPD.

**Discussion:**

Our Bayesian and correlation analyses support new dimensional conceptualizations of personality disorders.

## Highlights

• Neural correlates of posttraumatic stress disorder with personality disorder are unknown.
• We used frequentist and Bayesian statistical methods.
• We found no brain activation differences between different personality disorders.
• We found transdiagnostic associations between brain activation and clinical data.

## 1 Introduction

Post-traumatic stress disorder (PTSD) is a debilitating disorder that may occur after experiencing a traumatic event and is characterized by re-experiencing, avoidance, negative changes in mood and cognitions and hyperreactivity (American Psychiatric Association, 2013). PTSD has a lifetime prevalence of around 4 % (Koenen et al., 2017). There is a high comorbidity between PTSD and personality disorders, with more than a third of PTSD patients also fulfilling the diagnosis for a personality disorder (Friborg et al., 2013), such as borderline personality disorder (BPD, comorbidity 22 %) or cluster C, i.e. avoidant, dependent and obsessive–compulsive personality disorders (CPD; comorbidity 8–23 %). Conversely, more than 45 % of individuals with a personality disorder suffer from a comorbid PTSD (Massaal-van der Ree et al., 2022) and specifically, more than 50 % of people with BPD has a lifetime PTSD diagnosis (Scheiderer, Wood, & Trull, 2015).

Imaging studies have shown dysfunction in brain areas associated with emotion processing and regulation both in PTSD and borderline personality disorder. The amygdala is typically associated with emotion processing and hyperactive in people with PTSD upon being exposed to trauma-related and emotionally charged stimuli (Pitman et al., 2012, Stark et al., 2015). In BPD findings are more heterogeneous, with one *meta*-analysis showing lower activity in the amygdala and associated networks during tasks with a negative emotion versus neutral condition (Ruocco et al., 2013), while another more recent *meta*-analysis (Schulze, Schmahl, & Niedtfeld, 2016) showing higher left amygdala activity. Possible explanations are the heterogeneity of the samples and the suppressing effect of psychotropic medication on amygdala activity in medicated participants (Schulze, Schmahl, & Niedtfeld, 2016). Also, severity of state dissociation during scanning – that is associated with blunting amygdala activation - might have contributed to variation in amygdala activation (Lanius et al., 2010). For CPD, functional neuroimaging studies are scarce, although one study showed higher amygdala activation in CPD participants compared to controls in the anticipation phase during a reappraisal task (Denny et al., 2015).

Next to the amygdala, both BPD and PTSD participants showed hypoactivation of the posterior right insula and left postcentral gyrus compared to control participants and hyperactivation of the right middle frontal gyrus and left inferior frontal gyrus showed hyperactivation (Schulze et al., 2019). In one study, both BPD and CPD participants showed lower activation in the dorsal ACC during habituation to negative images compared to controls (Koenigsberg et al., 2014). The dlPFC is involved in effortful regulation and executive functions (Phillips et al., 2003) and seems to be hyperactive in PTSD participants and hypoactive in participants with BPD, compared to controls (Schulze et al., 2019). =The activation pattern in these brain areas in participants with both PTSD and a comorbid personality disorder is yet unknown.

Emotion dysregulation and dissociation are symptoms of both PTSD and BPD (American Psychiatric Association, 2013, Ford and Courtois, 2021).). Dissociation involved disruptions in neurological, psychological and cognitive functions (Lanius et al., 2010, Scalabrini et al., 2020), A recent meta-analysis identified brain areas from the emotion regulation network that were related to dissociation across the dissociative symptom spectrum; lower limbic activation in the amygdala, parahippocampus and insula, and higher activation in the hippocampus, cingulate cortex and medial frontal gyrus were found to be related to dissociative symptoms (Cavicchioli et al., 2023).

We studied brain activation during emotional processing in participants with PTSD and a comorbid BPD and/or CPD. The main research question was whether the brain response in areas involved in emotional processing during an emotional face task is influenced by type of comorbid personality disorder (BPD, CPD or both BPD and CPD) in PTSD participants. First, we focused our analyses on regions of interest (ROI) related to emotion processing in PTSD: i.e. bilateral amygdala, right insula, right dlPFC and right dmPFC. We expected higher activation of the amygdala in all PTSD + personality disorder groups compared to control participants and lower activation in the insula, dlPFC and dmPFC. Little is known about the neurobiological underpinnings of CPD. However, since both BPD and CPD are associated with impairment and poor functioning (Skodol et al., 2002) and the combination of BPD + CPD is associated with a higher prevalence of a comorbid anxiety disorder (Massaal-van der Ree et al., 2022), we hypothesized that PTSD participants with both comorbid BPD and CPD show more dysfunction than participants with PTSD + BPD or PTSD + CPD compared to controls, i.e. higher activation in the amygdala and lower activation in insula, dlPFC and dmPFC. The second research question is how brain activation relates to clinical measures in all PTSD participants together (transdiagnostically). We studied PTSD severity, emotion regulation problems, anger, dissociation, borderline symptom severity and depressive symptom severity. We hypothesized higher task-related activation in the amygdala, dlPFC and dmPFC, and lower activation in the insula to be related to higher PTSD severity, anger, emotion regulation problems and borderline symptoms. We expected lower amygdala activation to be related to more severe dissociation. This study was preregistered on the open science framework, ​(Aarts et al., 2022).

## 2 Methods

### 2.1 Participants

Participants in this study were part of two larger randomized controlled trials in the PROSPER-study, see also Snoek et al., 2020, van den End et al., 2021. Inclusion criteria were at least three traits of avoidant or obsessive–compulsive personality disorder, and/or four traits of dependent or BPD (i.e. one trait under the cutoff score for the classification); age between 18 and 65 years, sufficient understanding of the Dutch language and ability to provide written informed consent. Exclusion criteria were severe outward aggression, addiction or eating disorders interfering with treatment, current psychosis, mental retardation, a primary personality disorder other than BPD or CPD, benzodiazepine use exceeding three times 10 mg oxazepam equivalent per day. Other psychotropic medication needed to be stable for at least three weeks prior to study participation. In this study, we used a subset of data of participants from the PROSPER-trials that agreed to the scanning sessions, see Aarts et al. (2021). We recruited 88 participants with PTSD for the current study. Control participants were recruited through hersenonderzoek.nl (https://www.hersenonderzoek.nl), an online registry where you can match characteristics of your sample to control subjects. An additional exclusion criterion for control participants was any current diagnosis for a mental disorder. The 30 included control participants were matched to the patient groups’ distribution of age, sex and education level. The study was approved by the ethics committee of VU Medical Center.

### 2.2 Procedure

We screened participants for the PROSPER trial with the Structured Clinical Interview for DSM-5 Personality Disorders (SCID-5-PD), a semi-structured interview to assess personality disorders (Arntz et al., 2017, First et al., 2016). Based on the SCID-5-PD, we included participants in the PTSD + BPD trial or the PTSD + CPD trial, see Aarts et al. (2021). For the current study, we classified participants who fulfilled full criteria for both BPD and CPD in the PTSD + BPD + CPD group. All participants were asked to participate in the magnetic resonance imaging (MRI) part of the study. We then screened them with an MRI safety checklist and planned an appointment for the scanning session before participants’ treatment started. Prior to the scanning session, participants filled out a medication list to assess medication use in the previous 24 h. In a separate appointment, there was a clinical interview to assess PTSD symptoms and participants filled out questionnaires online. The full scanning protocol can be found in Aarts et al. (2021). Control participants came to the location once for the MRI scan and two neuropsychological tests.

### 2.3 Clinical measures

PTSD severity was measured with the Clinician-Administered PTSD Scale for DSM-5 (CAPS-5), a semi-structured interview that can be used to diagnose PTSD and gives a severity score with a total range between 0 and 80 (Boeschoten et al., 2014, Weathers et al., 2018). We assessed a range of clinical symptoms with questionnaires. Problems in emotion regulation were measured with the Difficulties in Emotion Regulation Scale (DERS, range 41–205; Gratz and Roemer (2004)). Anger was measured by the State Trait Anger Scale (STAS; Kroner and Reddon, 1992, Van der Ploeg et al., 1982). For this study, we used only the 10 items assessing trait anger (range 10 – 40). Dissociative symptoms were measured with the Dissociative Experiences Scale (DES, range 0–100; Bernstein, 1986, Van IJzendoorn et al., 1996. Borderline symptoms were measured with the Personality Assessment Inventory-Borderline Features Scale (PAI-BOR, range 0–72; Distel et al., 2009, Morey, 1991). A PAI-BOR score of 38 or higher is indicative of BPD. Depressive symptoms were measured with the Beck Depression Inventory (BDI, range 0–60; Beck et al., 1996, Upton, 2013). Finally, we asked participants to rate their tension level before and after the task, on a scale from 0 to 100.

### 2.4 Emotional faces task

**Fig. 1:**
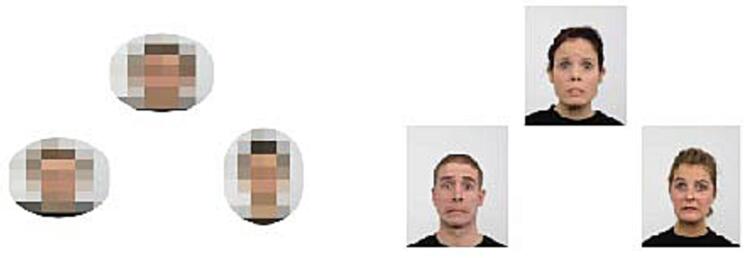
Example trials from the emotional faces task Scrambled faces trial (left) and fearful faces trial (right).

Brain activation was measured with functional MRI (fMRI) during an emotional face paradigm (adapted from Frijling et al. (2016)) and consisted of four conditions: fearful, angry, neutral and scrambled faces. Participants saw three faces at the same time and had to match the sex of the top picture to one of the bottom two. In the scrambled condition, participants had to match the orientation of the frame of the top picture to either one on the bottom. See Fig. 1 for picture examples. The task consisted of six pseudo-randomized blocks of neutral or scrambled faces, and five blocks of angry or fearful faces. Each block consisted of four trials. Our main contrast of interest was the fearful faces vs scrambled faces, secondary contrasts are angry vs scrambled and neutral vs scrambled. See Aarts et al. (2021) for more details. Task performance was calculated as proportion of correct responses.

### 2.5 MRI acquisition and preprocessing

Full imaging parameters of the task and scans are described in Aarts et al. (2021). Briefly, the scans were acquired on a GE Discovery MR750 3-Tesla scanner (General Electric, Milwauki, WI, USA) with a 32-channel head coil. First, an anatomical T1 scan was acquired (TR = 6.9 ms, TI = 900 ms, TE = 3 ms, flip angle = 9°, 168 slices, slice thickness 1 mm, matrix size 256x256). For the functional MRI scans, we acquired blip-up, blip-down scans with opposite phase encoding directions with the same field-of-view to correct for susceptibility induced distortions and high order shimming to homogenize the B_0_ magnetic field during the functional scans. We manually stopped the task scan after the task ended, around 205 volumes were acquired (TR = 2200 ms, TE = 26 ms, flip angle = 80°, 42 slices, slice thickness = 3 mm, matrix size = 64x64, 3.3 x 3.3 in plane resolution).

We processed functional images through the ‘fmriprep’ pipeline (version 21.0.1; Esteban et al., 2019; see supplements for full boilerplate) and smoothed by applying an 8 mm full-width half-maximum smoothing kernel and first-level analyses in Statistical Parametric Mapping 12 (SPM 12; Wellcome Trust Centre for Neuroimaging, London, UK).

MRI acquisition quality was checked in several steps. First, we removed participants who did not have a complete task scan because of technical problems. Then, we (visually) inspected all functional imaging data, for example for movement artefacts and ghosting. To further check the quality of T1 and fMRI sequences, the MRI Quality Control Tool (MRI QC; Esteban et al., 2017) and reports from fmriprep were used. We calculated quality parameters such as framewise displacement for fMRI sequences and used them to assess whether the quality of a scan was acceptable. We excluded scans with a mean framewise displacement of more than 0.5 mm from the analyses (Power, Schlaggar, & Petersen, 2015).

### 2.6 Statistical analyses

We preregistered our analysis on the Open Science Framework, see osf.io/9842Q. The main contrast of interest in the emotional face task was the fearful faces versus scrambled faces. Task activation for all participants are shown in Supplementary Fig. 1. We based the coordinates for our regions of interest (ROIs) for the main analysis on the *meta*-analysis by Schulze et al. (2019). These ROIs included the bilateral amygdala, right dlPFC (36, 40, 24), right dmPFC (2, 64, 20) and the right insula (36, −4, 8). For the subcortical areas (i.e. the amygdala) we used the Automated Anatomical Labeling (AAL) atlas. For the cortical areas (insula, dlPFC, dmPFC), we constructed 5 mm spherical ROIs around the specified coordinates. The activation levels for our main contrast of interest, fearful versus scrambled faces, were extracted from the ROIs and compared between participants with PTSD + CPD, PTSD + BPD, PTSD + BPD + CPD and control participants.

For the main research question we used an Analysis of CoVariance (ANCOVA) with diagnosis as independent variable, task contrast parameter as dependent variables and age and sex as covariates. We used a planned simple contrast with the control participants as the reference group. We checked bootstrapped results with 1000 permutations in case of violated assumptions for the ANCOVAs. We used a D/AP-SIDAK correction for multiple analyses across the ROIs. This correction uses the mutual correlation *r* between outcomes (Sankoh, Huque, & Dubey, 1997). These were computed using the online tool on https://www.quantitativeskills.com/sisa/calculations/bonhlp.htm. For our second research question, we ran a non-parametric Spearman’s correlation analysis on the brain activation in the specified ROIs with the seven above-mentioned clinical measures. We checked clinical data for outliers, but did not remove clinical data unless there was an invalid value or measurement error.

In addition, we ran the following secondary analyses. First, we did two types of sensitivity analyses with regard to medication status (as measured by use in last 24 h): a) the ANCOVAs with medication status as a covariate and b) subgroup comparisons of brain activation in all medicated PTSD participants vs. controls and in all non-medicated PTSD participants vs. controls. Second, we repeated the ANCOVAs on the contrasts angry vs. scrambled faces and neutral vs. scrambled faces. Third, we used Bayesian multilevel modeling to compare all groups to each other on the fearful vs. scrambled contrast. Bayesian modeling incorporates all outcomes into one integrative model, instead of fitting separate models for each ROI as in standard null-hypothesis significance testing (Chen et al., 2019). Bayesian analysis calculates a posterior distribution and the positive posterior probability (represented in a P + value). The further away the median of the distribution is from zero, the larger the effect and the larger the area under the curve (i.e. P + value), the more evidence for that effect. P + values that are either high (e.g. > 0.975) or low (e.g. < 0.025) provide very strong support for the effect, values > 0.95 or < 0.05 provide strong support and values > 0.90 or < 0.10 moderate support. In this Bayesian analysis, we included all ROIs from the main analyses, now bilaterally (amygdala, dlPFC, dmPFC, insula), plus the hippocampus (defined by the AAL atlas), superior occipital gyrus (±24, −96, 14; coordinates from Schulze et al., 2019), vmPFC (±4,41,-8; coordinates from Hayes, Hayes and Mikedis (2012)) and dorsal ACC (±18,40,17; Hayes, Hayes and Mikedis (2012)). Finally, to check for differences in regions not included in earlier analyses, we ran an exploratory whole-brain analyses with *p* <.001, uncorrected and a minimum extent cluster threshold of 5 voxels using SPM12.

## 3 Results

### 3.1 Sample

**Fig. 2:**
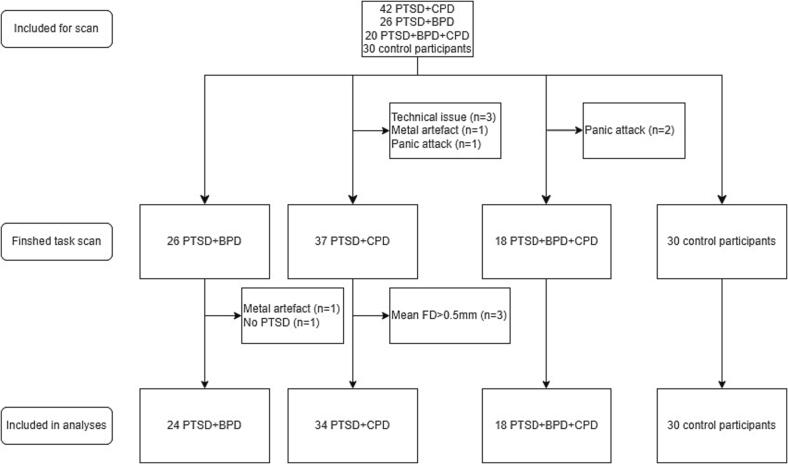
Flowchart of Participants.

After applying exclusion criteria, the final sample consisted of 76 participants with PTSD and comorbid personality disorder (34 PTSD + CPD, 24 PTSD + BPD, 18 PTSD + BPD + CPD), and 30 control participants (see flowchart in Fig. 2). Table 1 shows the demographic information and scores on the clinical measures per group. There were no significant differences between groups in age, sex, PTSD severity and task performance, but there were statistically significant between-group differences in emotion regulation, depression, anger, dissociation, borderline symptoms, tension before the task and tension after the task. Games-Howell corrected post-hoc tests showed significantly higher emotion regulation problems, anger scores, depression severity, dissociation severity and borderline symptom severity in participants with PTSD + BPD + CPD compared to PTSD + CPD, and higher anger scores and borderline symptom severity in PTSD + BPD than PTSD + CPD. An overview of the medication/psychoactive substances used in the 24 h before the scan is shown in Supplementary Table 1. Medication use did not differ significantly between groups (*χ^2^*(2) = 0.997, *p* =.607).

**Table 1:** Demographic Information and Clinical Measures (total N = 106).

	PTSD + CPD			PTSD + BPD			PTSD + BPD + CPD			Control participants			Test statistic (df)	p
	n	M	SD	n	M	SD	n	M	SD	n	M	SD		
Age	34	38.97	12.76	24	36.42	10.27	18	39.33	7.63	30	40.43	12.97	*F*(3,102) = 0.552	0.648
Sex (% f)		70.6 %			83.3 %						76.7 %		χ^2^(3, N = 106) = 1.297	0.730
CAPS-5	34	40.44	10.98	24	41.63	10.95	18	45.72	9.82	N/A	N/A	N/A	F(2,73) = 1.460	0.239
DERS	33	113.67	16.97	19	120.89	23.72	14	130.43	21.41	N/A	N/A	N/A	F(2,63) = 3.524	0.035*
BDI	33	32.03	11.96	21	31.52	12.77	15	40.67	9.98	N/A	N/A	N/A	F(2,66) = 3.286	0.044*
STAS	33	16.39	6.21	19	22.37	7.63	14	25.86	6.10	N/A	N/A	N/A	F(2,31.25) = 12.659	<0.001**
DES	23	15.48	10.29	18	22.06	15.19	14	37.47	21.98	N/A	N/A	N/A	F(2;25.975) = 6.491	0.005**
PAIBOR	33	34.67	8.40	19	40.16	6.73	14	44.36	5.18	N/A	N/A	N/A	F(2,63) = 9.110	<0.001**
Distress rating before task	16	29.44	25.08	19	40.68	26.94	14	43.21	27.92	29	21.07	23.40	F(3,74) = 3.470	0.020*
Distress rating after task	11	33.64	28.99	9	40.33	26.23	11	39.36	19.66	29	19.94	19.03	F(3,56) = 3.304	0.027*
Task performance	34	0.78	0.25	24	0.84	0.22	18	0.80	0.25	30	0.89	0.08	F(3,102) = 1.549	0.207

Note. PTSD = posttraumatic stress disorder, BPD = borderline personality disorder, CPD = cluster C personality disorder, BDI = Beck Depression Inventory, CAPS-5 = Clinician-Administered PTSD Scale for DSM-5, DES = Dissociative Experiences Scale, DERS = Difficulties in Emotion Regulation Scale, PAIBOR = Personality Assessment Inventory-Borderline Features Scale, STAS = State Trait Anger Scale.

* p <.05, ** p <.01.

### 3.2 Ancovas

**Fig. 3:**
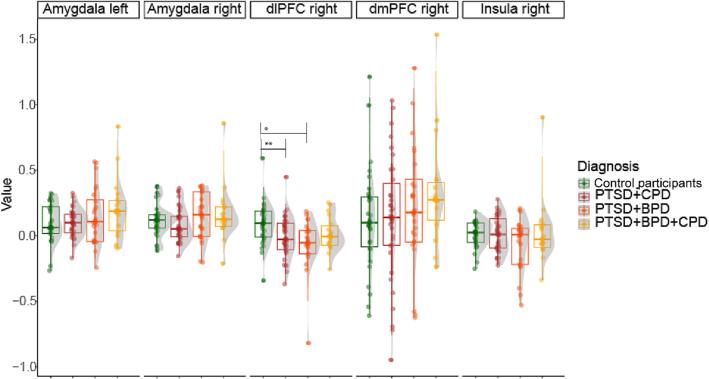
Raincloud Plots for Activation in the ROIs in the Fearful > Scrambled Faces Contrast*. Note.* BPD = borderline personality disorder, CPD = cluster C personality disorder, PTSD = posttraumatic stress disorder, dmPFC = dorsomedial prefrontal cortex, dlPFC = dorsolateral prefrontal cortex. ** *p* <.01; not significant when corrected for medication status.

Table 2 shows the results of the ROI analysis. For the fearful > scrambled faces contrast, results showed a significant difference between groups in the right dlPFC (*F*(3,100) = 4.200, *p* =.008) that was driven by lower activation in both PTSD + BPD (95 % confidence interval [0.062–––0.267], *p =*.002) and PTSD + CPD groups (95 % confidence interval [0.023–––0.183], *p =*.019) compared to control participants. The other contrasts (anger > scrambled and neutral > scrambled) revealed no significant differences between the groups. When controlling for medication status, the between-group difference in activation in the right dlPFC was no longer significant (right dlPFC *F*(3,91) = 1.773, *p* =.178). Fig. 3 shows the task-related activation for all groups in all ROIs.

**Table 2:** ROI Analysis of Covariance for the Effect of Diagnosis on Brain Activation.

Region of interest	*F* (3, 100)	*p*
Contrast fear > scrambled		
Left amygdala	1.634	0.186
Right amygdala	1.133	0.339
Right dmPFC	0.981	0.405
Right dlPFC	4.200	0.008**
Right insula	1.711	0.170
Contrast anger > scrambled		
Left amygdala	0.662	0.577
Right amygdala	0.461	0.710
Right dmPFC	0.921	0.434
Right dlPFC	0.804	0.372
Right insula	1.007	0.393
Contrast neutral > scrambled		
Left amygdala	0.471	0.704
Right amygdala	0.155	0.926
Right dmPFC	1.799	0.152
Right dlPFC	2.069	0.109
Right insula	1.601	0.194

*Note.* dmPFC = dorsomedial prefrontal cortex, dlPFC = dorsolateral prefrontal cortex.

** *p <.*017 (Sidak’s correction through D/AP method).

For the fearful > scrambled contrast among all PTSD participants, severity of dissociation showed a statistically significant negative association with activation in the right insula (*r*(53) = -0.35, *p* <.01) and right dmPFC (*r*(53) = -0.28, *p* <.05). Emotion regulation problems showed a statistically significant negative association with right dmPFC activation (*r*(64) = -0.26, *p* <.05). Other clinical measures did not show statistically significant correlations with brain activation, see Table 3 for all correlations.

**Table 3:** Spearman Correlations for Brain Activation in Fearful > Scrambled Contrast and Clinical Measures.

	Left amygdala	Right amygdala	Right dmPFC	Right dlPFC	Right insula	Emotion regulation problems	PTSD severity	Anger	Dissociation severity	Borderline severity	Depression severity	Tension before scan	Tension after scan
Left amygdala	–	–	–	–	–	–	–	–	–	–	–	–	–
Right amygdala	0.734** (n = 106)	–	–	–	–	–	–	–	–	–	–	–	–
Right dmPFC	0.364** (n = 106)	0.336** (n = 106)	–	–	–	–	–	–	–	–	–	–	–
Right dlPFC	0.155 (n = 106)	0.265** (n = 106)	0.072 (n = 106)	–	–	–	–	–	–	–	–	–	–
Right insula	0.300** (n = 106)	0.308** (n = 106)	0.179 (n = 106)	0.364** (n = 106)	–	–	–	–	–	–	–	–	–
Emotion regulation problems	-0.127 (n = 66)	0.023 (n = 66)	-0.256* (n = 66)	-0.049 (n = 66)	-0.142 (n = 66)	–	–	–	–	–	–	–	–
PTSD severity	-0.053 (n = 76)	-0.005 (n = 76)	-0.018 (n = 76)	0.025 (n = 76)	-0.082 (n = 76)	0.329** (n = 66)	–	–	–	–	–	–	–
Anger	0.017 (n = 66)	0.096 (n = 66)	-0.131 (n = 66)	-0.187 (n = 66)	-0.191 (n = 66)	0.388** (n = 66)	0.265* (n = 66)	–	–	–	–	–	–
Dissociation severity	-0.206 (n = 55)	-0.078 (n = 55)	-0.276* (n = 55)	-0.069 (n = 55)	-0.348** (n = 55)	0.410** (n = 55)	0.462** (n = 55)	0.596** (n = 55)	–	–	–	–	–
Borderline severity	0.038 (n = 66)	0.162 (n = 66)	-0.095 (n = 66)	-0.103 (n = 66)	-0.167 (n = 66)	0.574** (n = 66)	0.385** (n = 66)	0.637** (n = 66)	0.436** (n = 55)	–	–	–	–
Depression severity	-0.060 (n = 69)	0.033 (n = 69)	-0.196 (n = 69)	0.041 (n = 69)	-0.040 (n = 69)	0.601** (n = 66)	0.479** (n = 69)	0.369** (n = 66)	0.435** (n = 55)	0.524** (n = 66)	–	–	–
Distress rating before task	-0.005 (n = 78)	0.072 (n = 78)	-0.169 (n = 78)	-0.178 (n = 78)	0.149 (n = 78)	0.183 (n = 41)	0.256 (n = 49)	0.206 (n = 41)	0.348* (n = 40)	0.181 (n = 41)	0.428** (n = 43)	–	–
Distress rating after task	-0.019 (n = 60)	0.110 (n = 60)	0.048 (n = 60)	-0.116 (n = 60)	0.152 (n = 60)	-0.051 (n = 26)	0.333 (n = 31)	0.183 (n = 26)	0.498* (n = 25)	0.001 (n = 26)	0.326 (n = 27)	0.854** (n = 59)	–

*Note.* dmPFC = dorsomedial prefrontal cortex; dlPFC = dorsolateral prefrontal cortex; PTSD = posttraumatic stress disorder.

* *p* <.05; ** *p* <.01.

Medicated, as compared to unmedicated PTSD participants, showed higher brain activation in the fearful > scrambled contrast in the left amygdala (*t*(66) = -2.790, *p* =.007), right dmPFC (*t*(66) = -2.280, *p* =.026) and right insula (*t*(66) = -2.657, *p* =.010). There was no significant difference between medicated and unmedicated participants in the right amygdala (*t*(66) = -1.794, *p* =.077) and right dlPFC activation (*t*(66) = -0.989, *p* =.326, (see Supplementary Fig. 2 for a visualization). Medicated participants showed similar clinical characteristics as unmedicated participants, except for a lower borderline symptom severity (PAIBOR: *t*(58) = 2.036, *p* =.046) (see Supplementary Table 2).

### 3.3 Bayesian analysis

There was moderate to strong credible evidence for lower activation in the bilateral superior occipital cortex in participants with PTSD + BPD (P+ = 0.957 left and P+ = 0.947 right), and PTSD + CPD (P+ = 0.990 left and P+ = 0.976 right) compared to control participants, while there was very strong credible evidence for higher activation in these occipital areas in participants with PTSD + BPD + CPD compared with participants with PTSD + BPD (P+ = 1.000 left and right), participants with PTSD + CPD (P+ = 1.000 left and right) and control participants (P+ = 0.002 left and P+ = 0.004 right). There was moderate support for higher activation in the bilateral dmPFC in participants with PTSD + BPD + CPD than control participants (P+ = 0.067 and 0.073). Activation in all ROIs was higher in the participants with PTSD + BPD + CPD than both participants with PTSD + BPD and PTSD + CPD, with moderate to strong support for the difference in almost all ROIs. See Supplementary Table 3 for all mean differences, standard deviations and P + values, Supplementary Fig. 3 for boxplots of the activation in all brain areas from this analysis and Supplementary Figure 4–9 for the posterior distributions of all the contrasts.

### 3.4 Whole-brain analysis

After Family-Wise Error correction (p <.05), one area showed significant differences between the groups. In the left supramarginal gyrus, activation was higher in the PTSD + BPD + CPD group than the PTSD + BPD group (Montreal Neurological Institute coordinates (-44,-24,18; Z = 4.85, p_FWEcorr_ = 0.020, cluster size = 6). See Supplementary Table 4 for the full list of uncorrected findings from the whole-brain analyses.

## 4 Discussion

In this study, we investigated differences in brain activation during an emotional face task between participants with PTSD and comorbid CPD and/or BPD and with matched control participants. There were no significant differences in activation between the groups using ANCOVAs, when corrected for medication status. Bayesian analyses showed credible evidence for higher activation in almost all ROIs in the PTSD + BPD + CPD participants, which is the group with statistically significant more clinical symptoms, compared to the PTSD + BPD and PTSD + CPD groups. Transdiagnostically, i.e. across all PTSD groups, dissociation severity was negatively related to right insula and right dmPFC activation, and severity of emotion regulation problems was negatively related to right dmPFC activation.

It is remarkable that we did not observe a case-control difference in amygdala, dmPFC, insula and dlPFC (corrected for medication status) activation, despite this being a robust finding in PTSD and BPD literature (e.g. Schulze et al., 2019). It is possible that comorbid dissociation and depression symptoms clouded our results. Specifically, dissociation scores in our PTSD + BPD + CPD group appear to be higher than the scores in the samples used in a meta-analysis on amygdala activation in PTSD and BPD (Lyssenko et al., 2017). In general, dissociation has been related to lower amygdala and insula activation (see Lanius et al., 2010 for an overview). Furthermore, depression scores in our sample indicate severe depression (Upton, 2013) and activation in the amygdala, insula and middle frontal gyrus have been found to be lower in depressive patients than control participants or than participants with PTSD or BPD (Schulze et al., 2019). Finally, individuals with PTSD and comorbid personality disorders, as included in the present study, might use opposite neurobiological circuits during emotional processing and cancel each other out. In fact, participants with PTSD showed higher dlPFC activation compared with control participants, while this was lower in participants with BPD (Schulze et al., (2019).

All together, we did not find robust support for categorical differences between PTSD + BPD and PTSD + CPD participants, while symptom severity associates with specific brain activation patterns taking the categories together. The transdiagnostic negative association between emotion regulation problems and dmPFC activation is in line with earlier studies showing that the dmPFC is involved in reappraisal of negative emotions, with activation related to planning of a response to imminent threat and regulation of the amygdalar response (Etkin, Egner, & Kalisch, 2011). The negative association between dissociation severity and activation in the right dmPFC and right insula is also in line with the conceptualization of dissociation as a coping mechanism of overmodulation of emotions, where increased activation of prefrontal areas (dmPFC) associates with decreased activation of emotion processing areas (amygdala, insula) (Cavicchioli et al., 2023, Lanius et al., 2010). Although the negative association between dissociation and insula activation fits within this model, the negative association between dissociation and dmPFC activation does not. In a systematic review, Roydeva and Reinders (2021) describe higher dmPFC activation in dissociation across disorders and paradigms. In fact, the Bayesian analyses show support for higher activation in all ROIs (such as the amygdala, hippocampus, dlPFC, dmPFC) in participants with PTSD + BPD + CPD than participants with PTSD + BPD or PTSD + CPD. This is in line with new ways of conceptualizing personality disorders, such as in the latest revision of the International Classification of Diseases, where personality disorders are no longer classified categorically but are rated along a severity scale (Bach et al., 2022).

Strengths of this study are the participation of a rarely studied, clinically relevant group with comorbid PTSD, borderline and/or cluster C personality disorders. As far as we know, this is one of the first fMRI studies in this group. Another strength is the application of Bayesian analyses in fMRI research. The present study showed a difference in the findings between the case-control analyses and the Bayesian analyses, where the Bayesian analysis seems to be more sensitive to detect activation differences between the groups. An important advantage of a Bayesian over a classic ANOVA analysis, is that it integrates information from all ROIs, instead of analyzing them as separate entities and then later correcting for multiple testing (Chen et al., 2019). This makes the Bayesian analysis more efficient. Importantly, traditional statistical analyses give dichotomous results as the main outcome, giving rise to loss of a lot of information from the data. Bayesian analyses leave room for more dimensional and nuanced interpretations (Taylor et al., 2023).

Some limitations apply. First, we did not include a PTSD-only or a BPD/CPD-only control group. This could have helped interpretation of the findings by isolating the clinical symptoms and diagnoses of participants. Secondly, we did not include a measure for state dissociation during scanning. Evidence suggests that dissociation-related brain alterations in BPD may be best detected during acute dissociation in the scanner (Krause-Utz et al., 2021), while we measured trait dissociation outside of the scanner. Future studies could include an adapted version of the Response to Script Driven Imagery Scale (Hopper et al., 2007) in their scanning protocols to measure state dissociation. Thirdly, we do not have clinical measures for the control subjects. Although we would expect little variation and generally low scores in this group, it could be interesting to compare correlations between clinical and brain activation measures between PTSD and control subjects. Finally, we lack information about the indication for medication prescription. We unexpectedly found higher activation in the amygdala in the medicated participants when compared to unmedicated PTSD participants.

There are no differences on clinical measures or level of distress during the scan between these groups, except for lower borderline symptoms in the medicated group. Medication is not a first-line treatment for PTSD and only two types of medication are approved for use in PTSD (sertraline and paroxetine; see Burback et al., 2023 for an up-to-date overview of pharmacological intervention in PTSD). This means that participants in our sample could be prescribed specific medication ór for a comorbid (affective) disorder, ór for specific PTSD symptoms, making it more difficult to interpret our findings.

## 5 Conclusion

This is one of the first studies that investigates brain activation in participants with PTSD and a comorbid personality disorder, a group that is very prevalent in clinical practice. All in all, we did not find support for categorical differences between the PTSD + BPD and PTSD + CPD groups, but found significant associations between severity of clinical symptoms (transdiagnostically and in the PTSD + BPD + CPD group) and activation in the right insula and right dmPFC. Our findings fit in a transdiagnostic and dimensional approach to personality disorders.

## CRediT authorship contribution statement

**Inga Aarts:** Conceptualization, Investigation, Data curation, Formal analysis, Writing – original draft, Visualization. **Chris Vriend:** Conceptualization, Data curation, Formal analysis, Software, Supervision, Writing – review & editing. **Odile A. van den Heuvel:** Conceptualization, Supervision, Writing – review & editing. **Kathleen Thomaes:** Conceptualization, Funding acquisition, Supervision, Writing – review & editing.

## Declaration of competing interest

The authors declare that they have no known competing financial interests or personal relationships that could have appeared to influence the work reported in this paper.

## Data Availability

Data will be made available on request.
