# Supplementary material for: Brain activation during an emotional task in participants with PTSD and borderline and/or cluster C personality disorders
Source: Neuroimage Clin. 2023 Dec 18;41:103554. doi: 10.1016/j.nicl.2023.103554 (PMC10777111; doi:10.1016/j.nicl.2023.103554)
Supplement: Supplementary data 1 [file mmc1.docx]

**Supplementary information**

**Supplementary Figure 1**

*Main Task Effect for All Participants (N = 106)*

*
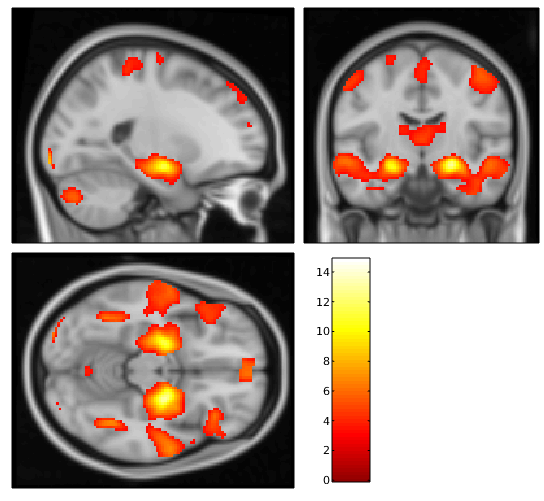
*

*Note.* Activation for all participants together in the fearful > scrambled contrast. Uncorrected results at *p* < .001.

**Supplementary Table 1**

*Overview of Medication and Other Psychoactive Substances Taken in the 24 Hours Before the Scan*

|  | PTSD+CPD  n | % | PTSD+BPD  n | % | PTSD+BPD+CPD  n | % |
| --- | --- | --- | --- | --- | --- | --- |
| No data | 3 | 8.8 | 2 | 8.3 | 3 | 16.7 |
| No medication | 18 | 52.9 | 10 | 41.7 | 7 | 38.9 |
| Medication | 13* | 38.2 | 12* | 50 | 8* | 44.4 |
| SSRI | 5 | 14.7 | 6 | 0.25 | 3 | 16.7 |
| SNRI | 1 | 2.9 | 0 | 0 | 1 | 4.2 |
| TCA | 4 | 11.8 | 0 | 0 | 1 | 4.2 |
| Bupropion | 0 | 0 | 0 | 0 | 2 | 8.3 |
| Unknown antidepressant | 0 | 0 | 0 | 0 | 1 | 4.2 |
| Benzodiazepine | 1 | 2.9 | 1 | 4.2 | 0 | 0 |
| Antipsychotic | 2 | 5.9 | 1 | 4.2 | 1 | 4.2 |
| Amphetamine | 0 | 0 | 1 | 4.2 | 0 | 0 |
| Other | 1 | 2.9 | 1 | 4.2 | 0 | 0 |
| Alcohol | 1 | 2.9 | 5 | 20.8 | 2 | 8.3 |
| Cannabis | 4 | 11.8 | 3 | 12.5 | 1 | 4.2 |

*Note.* PTSD = posttraumatic stress disorder; BPD = borderline personality disorder; CPD = cluster C personality disorder; SSRI = selective serotonin reuptake inhibitors; SNRI = serotonin and norepinephrine reuptake inhibitors; TCA = tricyclic antidepressant

* Participants might take more than one type of medication.

**Supplementary Figure 2**

*Raincloud Plots and Boxplots for Activation (Fearful > Scrambled Faces) in the ROIs in Unmedicated and Medicated PTSD Participants*


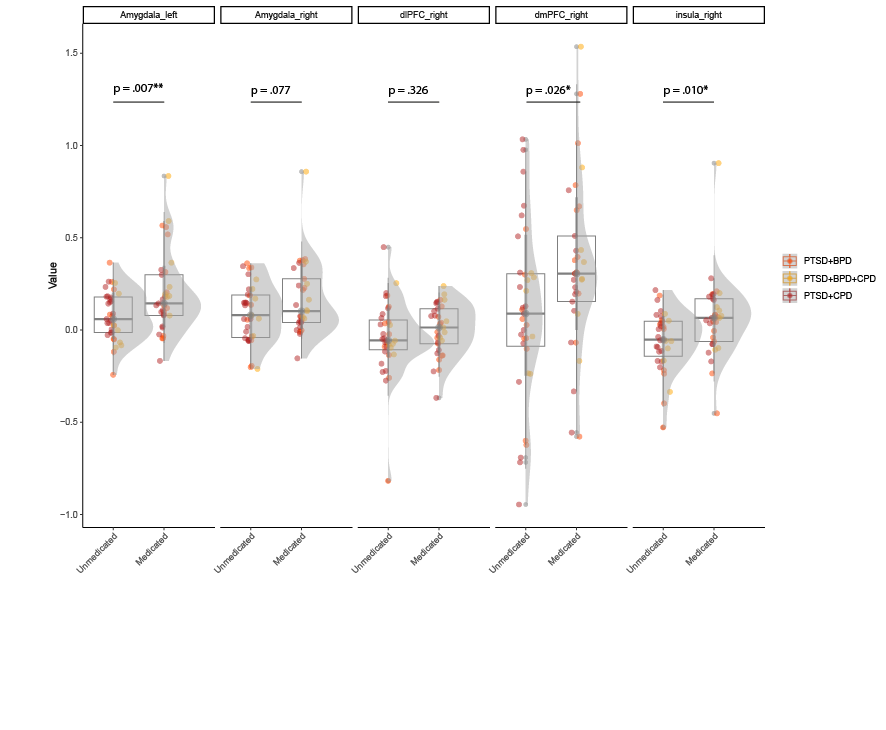


*Note.*; dlPFC = dorsolateral prefrontal cortex; dmPFC = dorsomedial prefrontal cortex; PTSD = posttraumatic stress disorder; BPD = borderline personality disorder; CPD = cluster c personality disorder.

* *p* < .01; ** *p* < .05

**Supplementary Table 2**

*Comparison of Clinical Measures in Medicated vs Unmedicated Patients*

|  | No medication | | | Medication | | | t (df) | *p* (two-sided) |
| --- | --- | --- | --- | --- | --- | --- | --- | --- |
|  | n | Mean | SD | n | Mean | SD |  |  |
| Age | 35 | 37.00 | 10.817 | 33 | 39.73 | 10.838 | -1.038(66) | .303 |
| Sex (%women) | 35 | 77.1% |  | 33 | 72.7% |  | χ^2^(1)=.177 | .674 |
| CAPS-5 | 35 | 43.51 | 11.65 | 33 | 40.30 | 9.84 | 1.224(66) | .225 |
| DERS | 32 | 122.28 | 23.39 | 28 | 114.54 | 17.58 | 1.433(58) | .157 |
| BDI | 32 | 35.09 | 13.81 | 31 | 31.26 | 10.47 | 1.239(61) | .220 |
| STAS | 32 | 21.69 | 8.05 | 28 | 18.29 | 7.02 | 223.00† | .098 |
| DES | 25 | 25.70 | 17.96 | 26 | 17.91 | 13.84 | 336.5† | .055 |
| PAIBOR | 32 | 40.09 | 8.73 | 28 | 35.71 | 7.81 | 2.036(58) | .046* |
| Distress rating before task | 25 | 39.92 | 23.088 | 23 | 37.00 | 30.130 | -0.379(46) | .353 |
| Distress rating after task | 16 | 44.38 | 23.157 | 14 | 32.36 | 24.450 | -1.382(28) | .089 |
| Task performance | 35 | .81 | .24 | 33 | .81 | .22 | .024 (66) | .981 |

*Note.* PTSD = posttraumatic stress disorder, BPD = borderline personality disorder, CPD = cluster C personality disorder, CAPS-5 = Clinician-Administered PTSD Scale for DSM-5, DERS = Difficulties in Emotion Regulation Scale, STAS = State Trait Anger Scale, DES = Dissociative Experiences Scale, PAIBOR = Personality Assessment Inventory-Borderline Features Scale, BDI = Beck Depression Inventory, dmPFC = dorsomedial prefrontal cortex; dlPFC = dorsolateral prefrontal cortex

† = Mann-Whitney U test

* = *p* < .05.

**Supplementary Table 3**

*Results from the Bayesian Analyses for All Group Comparisons*

|  | HC - PTSD+CPD | |  | HC - PTSD+BPD | |  | HC - PTSD+BPD+CPD | | | PTSD+BPD – PTSD+CPD | | | PTSD+BPD+CPD – PTSD+CPD | | | PTSD+BPD+CPD – PTSD+BPD | | |
| --- | --- | --- | --- | --- | --- | --- | --- | --- | --- | --- | --- | --- | --- | --- | --- | --- | --- | --- |
|  | mean | SD | P+ | mean | SD | P+ | mean | SD | P+ | mean | SD | P+ | mean | SD | P+ | mean | SD | P+ |
| Sup. occ. left | 0.152 | 0.068 | **0.990** | 0.119 | 0.073 | **0.957** | -0.246 | 0.080 | **0.002** | 0.033 | 0.081 | 0.660 | 0.398 | 0.092 | **1.000** | 0.366 | 0.094 | **1.000** |
| Sup. occ. right | 0.122 | 0.063 | **0.976** | 0.103 | 0.068 | **0.946** | -0.204 | 0.076 | **0.004** | 0.018 | 0.076 | 0.608 | 0.326 | 0.092 | **1.000** | 0.307 | 0.095 | **1.000** |
| Amygdala left | 0.016 | 0.047 | 0.632 | 0.013 | 0.047 | 0.612 | -0.064 | 0.053 | 0.110 | 0.003 | 0.053 | 0.515 | 0.080 | 0.064 | **0.901** | 0.077 | 0.057 | **0.911** |
| Amygdala right | 0.016 | 0.047 | 0.635 | 0.012 | 0.047 | 0.604 | -0.059 | 0.053 | 0.129 | 0.004 | 0.053 | 0.530 | 0.075 | 0.063 | 0.891 | 0.071 | 0.057 | 0.891 |
| dACC left | 0.007 | 0.045 | 0.567 | 0.023 | 0.044 | 0.701 | -0.056 | 0.052 | 0.135 | -0.016 | 0.049 | 0.369 | 0.063 | 0.061 | 0.858 | 0.080 | 0.055 | **0.923** |
| dACC right | 0.010 | 0.044 | 0.594 | 0.023 | 0.045 | 0.696 | -0.058 | 0.051 | 0.128 | -0.013 | 0.049 | 0.391 | 0.068 | 0.060 | 0.874 | 0.081 | 0.055 | **0.927** |
| dlPFC left | 0.009 | 0.046 | 0.583 | 0.029 | 0.045 | 0.747 | -0.063 | 0.051 | 0.104 | -0.020 | 0.050 | 0.340 | 0.072 | 0.060 | 0.887 | 0.093 | 0.055 | **0.956** |
| dlPFC right | 0.013 | 0.046 | 0.612 | 0.030 | 0.046 | 0.741 | -0.056 | 0.053 | 0.141 | -0.017 | 0.050 | 0.370 | 0.069 | 0.061 | 0.870 | 0.085 | 0.055 | **0.934** |
| dmPFC left | 0.024 | 0.052 | 0.680 | 0.003 | 0.051 | 0.540 | -0.084 | 0.059 | **0.067** | 0.021 | 0.058 | 0.631 | 0.108 | 0.072 | **0.939** | 0.087 | 0.064 | **0.918** |
| dmPFC right | 0.029 | 0.052 | 0.719 | 0.000 | 0.051 | 0.510 | -0.082 | 0.058 | **0.073** | 0.030 | 0.059 | 0.689 | 0.111 | 0.071 | **0.949** | 0.082 | 0.063 | **0.904** |
| Hippocampus left | 0.016 | 0.044 | 0.636 | 0.019 | 0.044 | 0.662 | -0.064 | 0.051 | 0.102 | -0.003 | 0.049 | 0.464 | 0.080 | 0.059 | **0.917** | 0.083 | 0.054 | **0.938** |
| Hipppcampus right | 0.015 | 0.044 | 0.628 | 0.018 | 0.045 | 0.660 | -0.064 | 0.051 | 0.106 | -0.003 | 0.049 | 0.461 | 0.079 | 0.060 | **0.910** | 0.082 | 0.055 | **0.930** |
| Insula left | 0.013 | 0.045 | 0.611 | 0.030 | 0.045 | 0.743 | -0.065 | 0.051 | 0.100 | -0.016 | 0.049 | 0.372 | 0.079 | 0.060 | **0.906** | 0.095 | 0.054 | **0.956** |
| Insula right | 0.007 | 0.045 | 0.566 | 0.029 | 0.045 | 0.745 | -0.061 | 0.052 | 0.119 | -0.022 | 0.050 | 0.324 | 0.068 | 0.062 | 0.867 | 0.090 | 0.055 | **0.946** |
| vmPFC left | -0.003 | 0.048 | 0.480 | 0.022 | 0.046 | 0.686 | -0.058 | 0.054 | 0.137 | -0.026 | 0.052 | 0.306 | 0.054 | 0.065 | 0.805 | 0.080 | 0.058 | **0.916** |
| vmPFC right | 0.001 | 0.047 | 0.515 | 0.024 | 0.045 | 0.708 | -0.060 | 0.053 | 0.123 | -0.024 | 0.051 | 0.325 | 0.061 | 0.064 | 0.833 | 0.085 | 0.057 | **0.931** |

*Note.* Mean represents the mean difference between the groups noted in the column above, calculated as first group – second group. P+ gives the probability for finding an effect is different from zero, values in bold are <.10 or >.90. A high P+ means probability for a positive effect (so difference larger than zero), a low P+ means probability for a negative effect (low probability for positive effect = high probability for negative effect). HC = healthy controls; PTSD = posttraumatic stress disorder; BPD = borderline personality disorder; CPD = cluster c personality disorder; Sup. occ. Superior occipital cortex; dACC = dorsal anterior cingulate cortex; dlPFC = dorsolateral prefrontal cortex; dmPFC = dorsomedial prefrontal cortex; vmPFC = ventromedial prefrontal cortex.

**Supplementary Figure 3**

*Boxplots for Activation in All ROIs from the Bayesian Analyses*


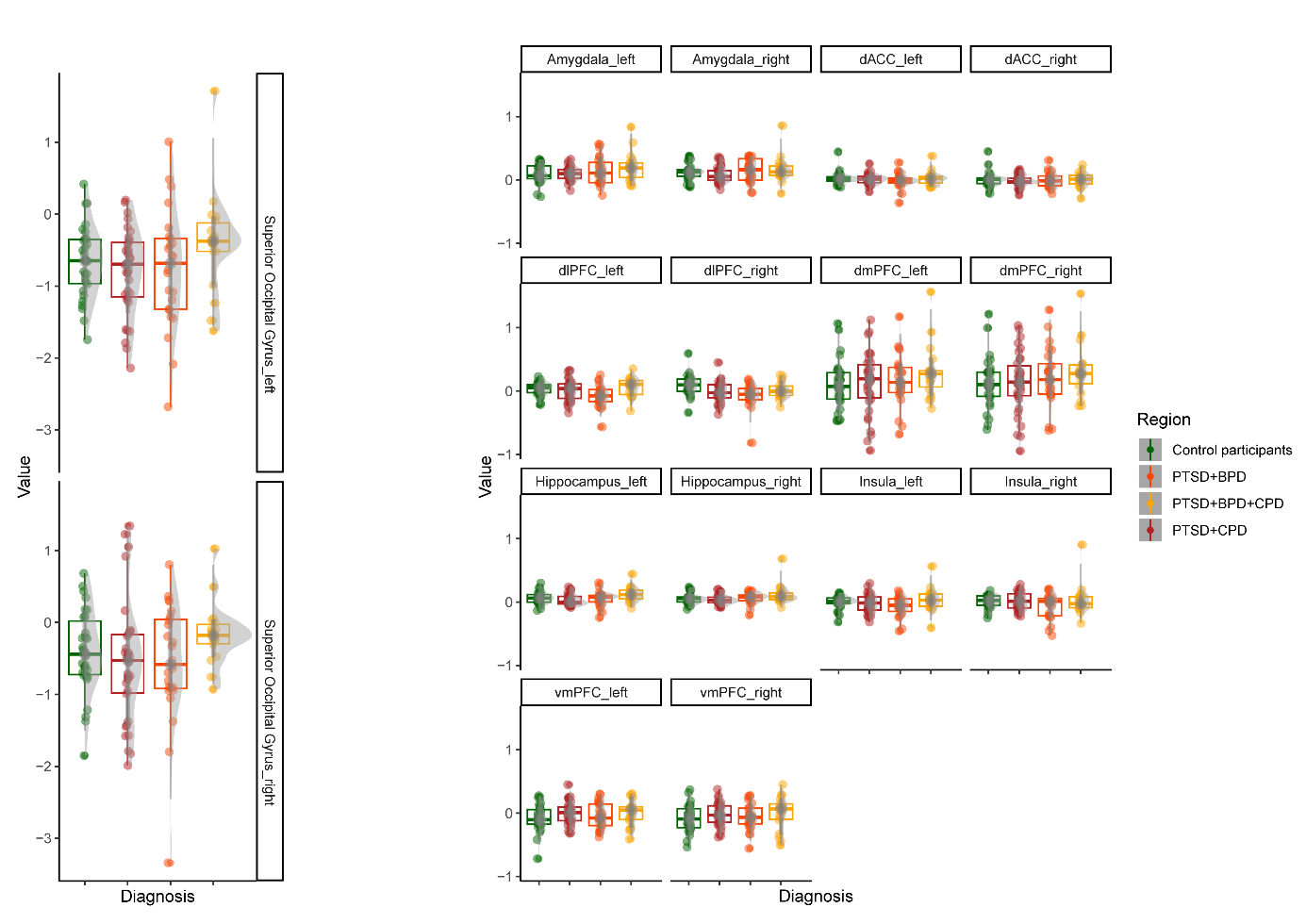


*Note.* PTSD = posttraumatic stress disorder; BPD = borderline personality disorder; CPD = cluster c personality disorder; dACC = dorsal anterior cingulate cortex; dlPFC = dorsolateral prefrontal cortex; dmPFC = dorsomedial prefrontal cortex; vmPFC = ventromedial prefrontal cortex.

**Supplementary Figure 4**

*Ridge Plot for the P+ Value in Control Participants vs Participants with PTSD+CPD*

*
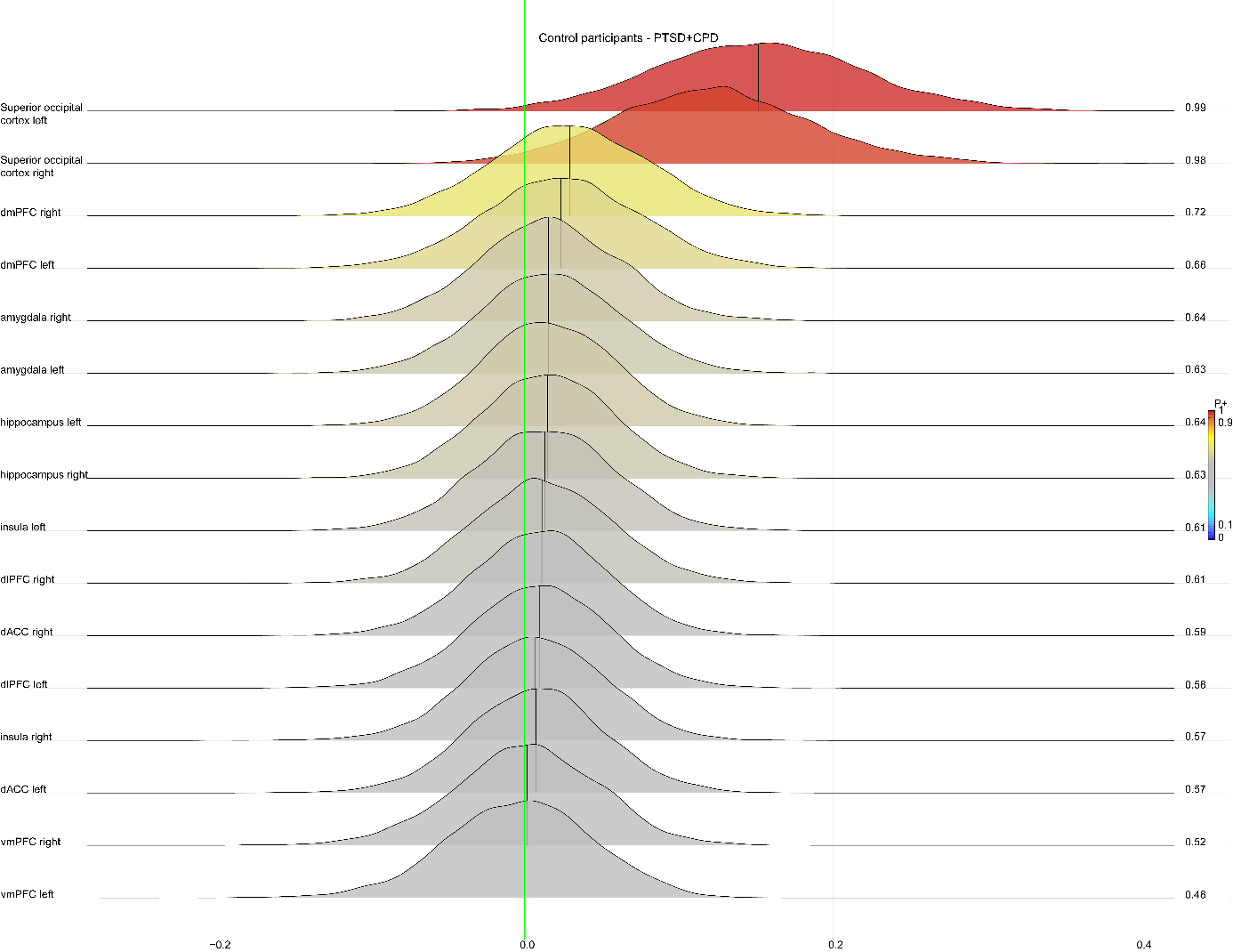
*

**Supplementary Figure 5**

*Ridge Plot for the P+ Value in* *Control Participants vs Participants with PTSD+BPD*

**
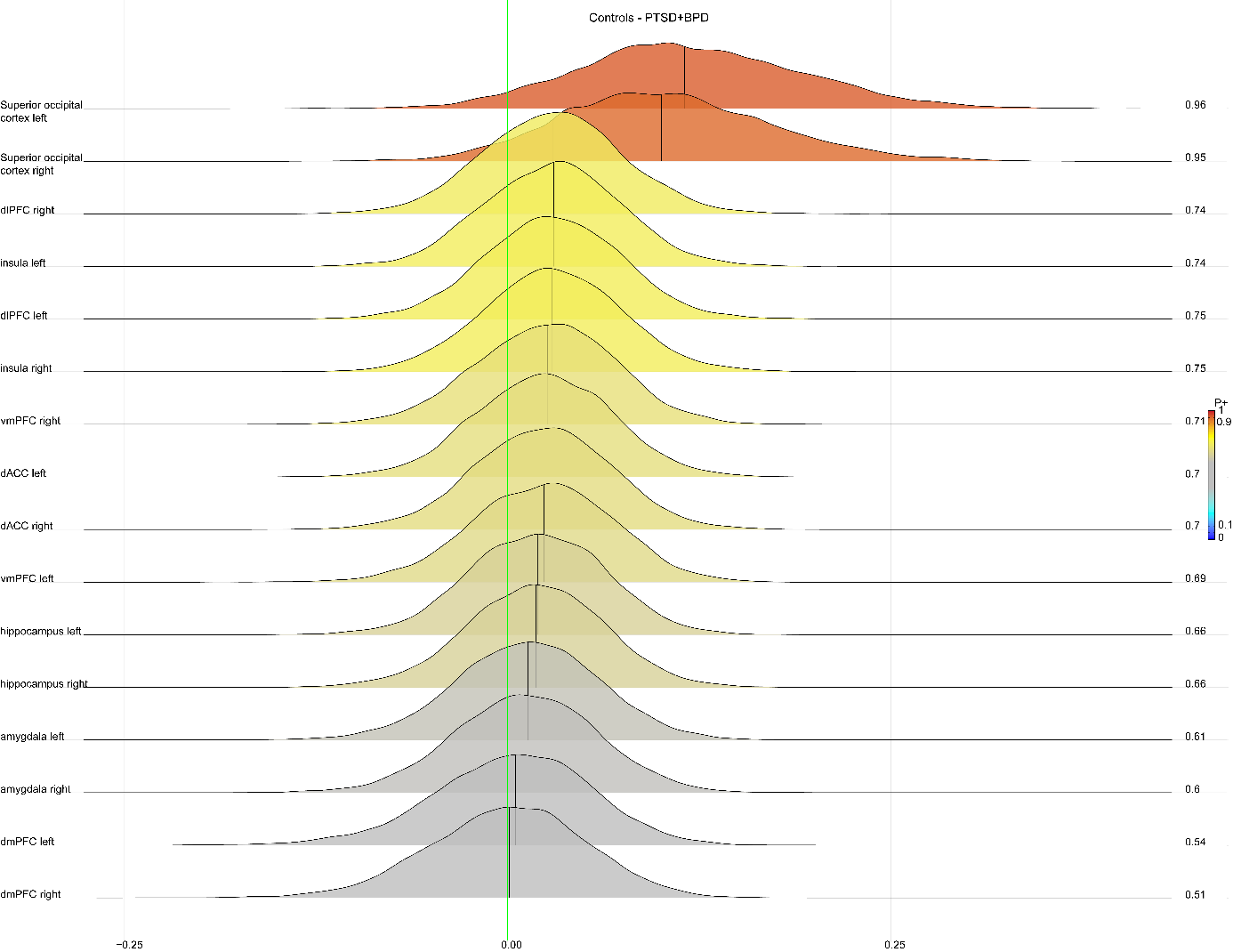
**

**Supplementary Figure 6**

*Ridge Plot for the P+ Value in Control Participants vs Participants with PTSD+BPD+CPD*

**
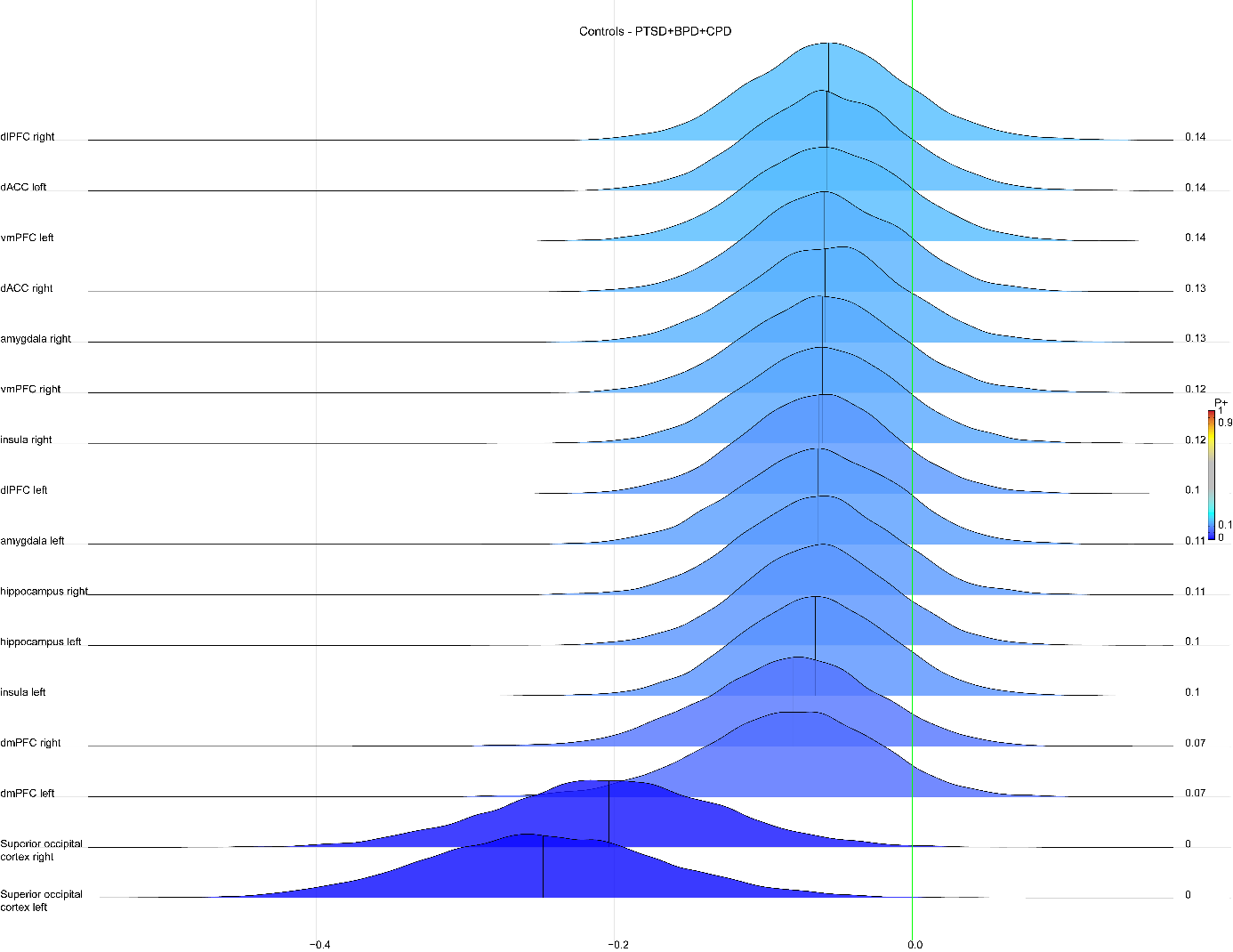
**

**Supplementary Figure 7**

*Ridge Plot for the P+ Value in Participants with PTSD+BPD+CPD vs Participants with PTSD+BPD*

**
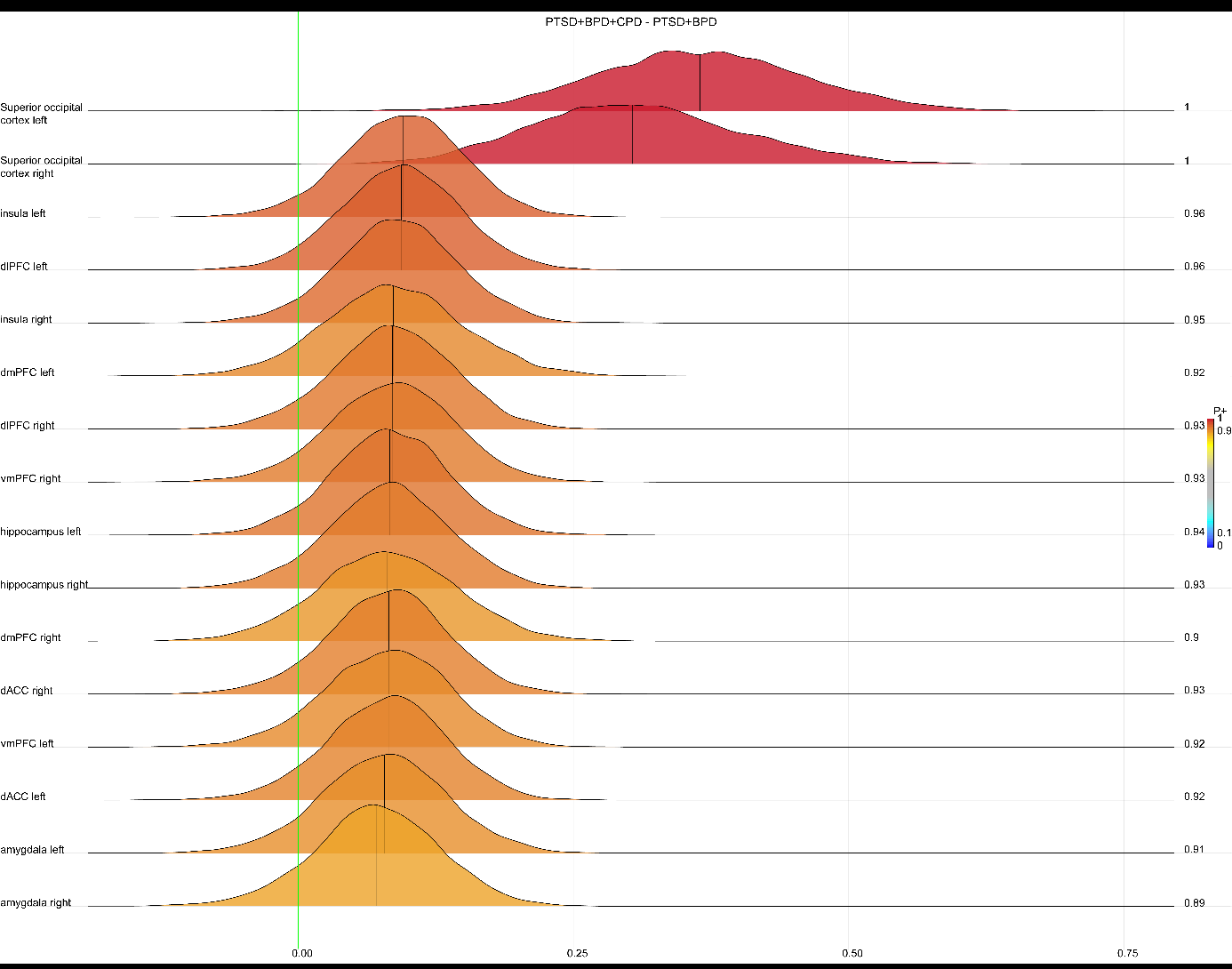
**

**Supplementary Figure 8**

*Ridge Plot for the P+ Value in* *Participants with PTSD+BPD+CPD vs Participants with PTSD+CPD*

**
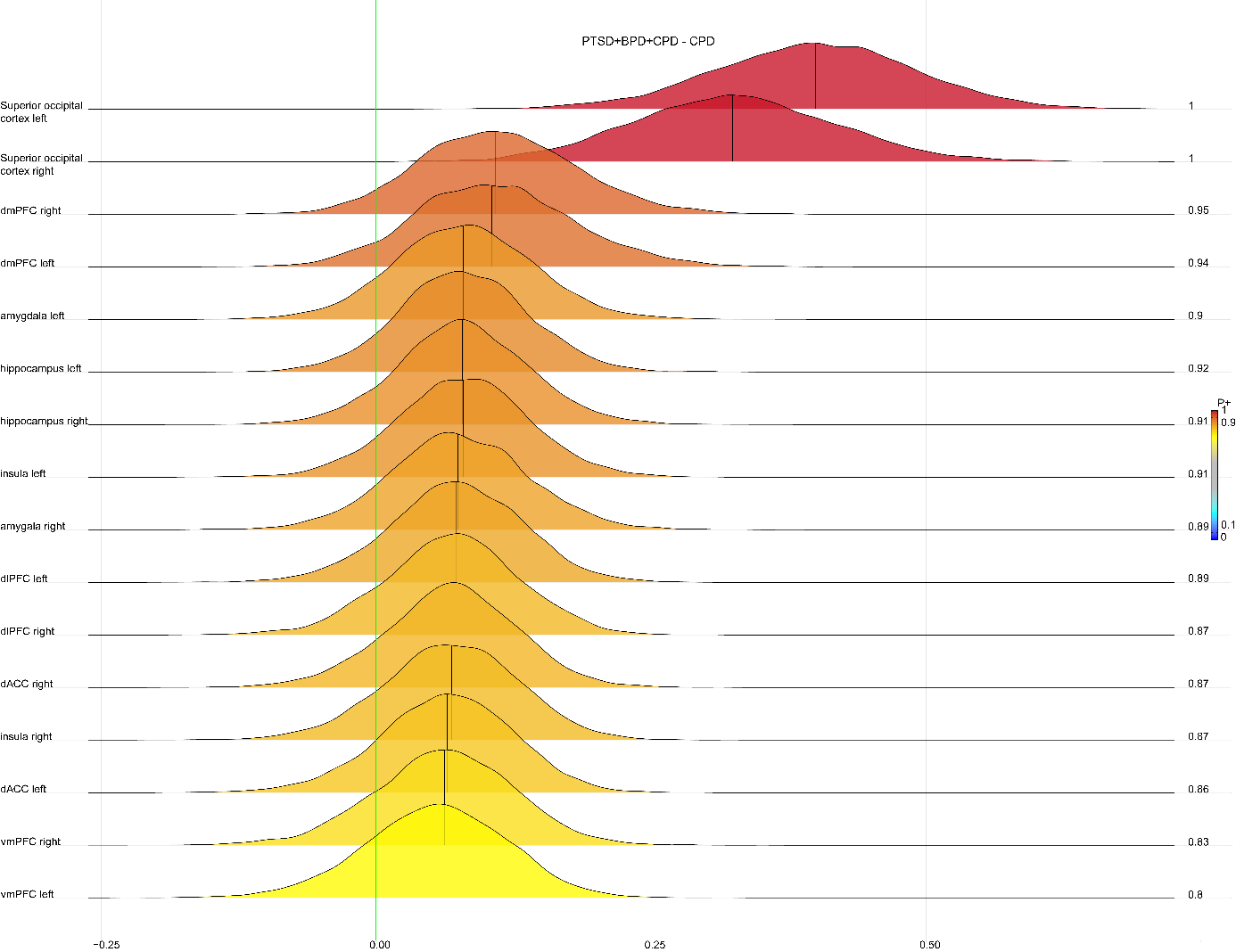
**

**Supplementary Figure 9**

*Ridge Plot for the P+ Value in Participants with PTSD+BPD vs Participants with PTSD+CPD*


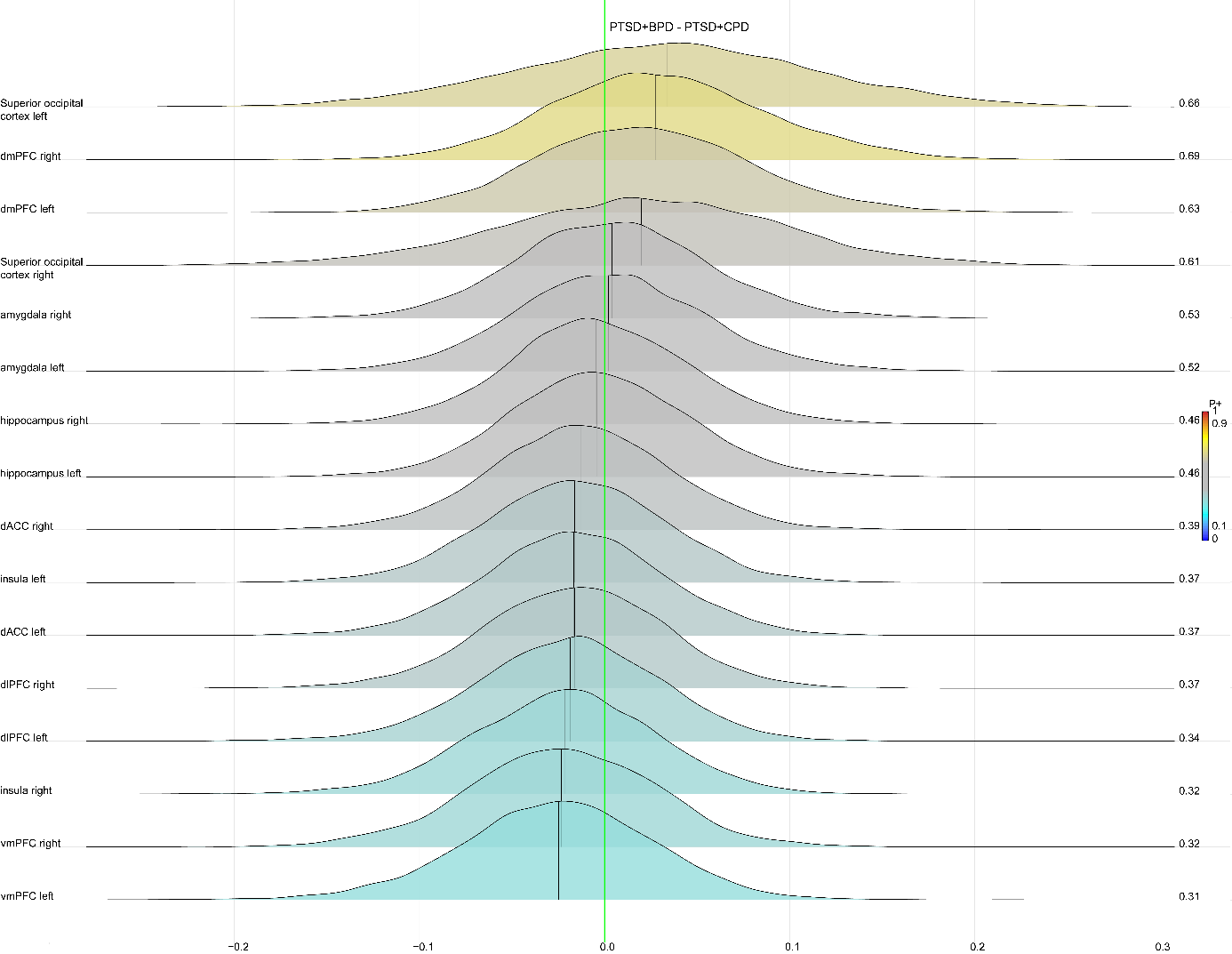


**Supplementary Table 4**

*Uncorrected Wholebrain Analysis with p<0.001 and Minimal Extent Cluster Size of 5*

| Brain region | Cluster size | Z-score | MNI coordinates | | |
| --- | --- | --- | --- | --- | --- |
|  |  |  | x | y | z |
| *HC > patients* | |  |  |  |  |
| Left supramarginal gyrus | 115 | 4.68 | -46 | -32 | 32 |
| Right superior parietal lobe | 55 | 4.03 | 28 | -46 | 34 |
| Right postcentral gyrus | 71 | 3.92 | 48 | -22 | 38 |
| Left thalamus | 110 | 3.77 | -2 | -10 | -2 |
| Left cerebellum | 15 | 3.65 | -26 | -64 | -42 |
| Left angular gyrus | 20 | 3.59 | -32 | -56 | 30 |
| Corpus callosum | 22 | 3.54 | 2 | 28 | 8 |
| Left cerebellum | 21 | 3.53 | -8 | -70 | -32 |
| Left anterior prefrontal cortex | 24 | 3.50 | -22 | 44 | 8 |
| Right cerebellum | 18 | 3.44 | 10 | -52 | -4 |
| Left supplementary motor area | 11 | 3.39 | -14 | 6 | 62 |
| Right middle frontal gyrus | 29 | 3.36 | 36 | 40 | 22 |
| Left cerebellum | 11 | 3.33 | -14 | -62 | -38 |
| Left parietal inferior cortex | 11 | 3.25 | -52 | -24 | 46 |
| Right primary visual cortex | 7 | 3.18 | 28 | -70 | 12 |
| *HC < Patients* | |  |  |  |  |
| No suprathreshold clusters | |  |  |  |  |
| *HC > PTSD+CPD* |  |  |  |  |  |
| Left cerebellum | 23 | 3.42 | -14 | -64 | -36 |
| Right anterior thalamus | 6 | 3.21 | 2 | -2 | -2 |
| *HC < PTSD+CPD* |  |  |  |  |  |
| No suprathreshold clusters | | | | | |
| *HC > PTSD+BPD* |  |  |  |  |  |
| Left supramarginal gyrus | 330 | 4.55 | -44 | -30 | 34 |
| Left precentral gyrus | 92 | 4.27 | -56 | 6 | 28 |
| Left anterior prefrontal cortex | 81 | 4.10 | -22 | 46 | 10 |
| Right superior parietal lobe | 62 | 4.01 | 30 | -46 | 34 |
| Right primary sensory cortex | 165 | 4.01 | 46 | -22 | 38 |
| Right middle cingulum | 117 | 3.86 | 4 | -14 | 2 |
| Left superior parietal lobe | 97 | 3.82 | -32 | -54 | 32 |
| Left primary visual cortex | 131 | 3.82 | -18 | -86 | 10 |
| Left cerebellum | 22 | 3.71 | -26 | -64 | -42 |
| Left superior parietal lobe | 28 | 3.68 | -28 | -42 | 48 |
| Left superior occipital cortex | 69 | 3.66 | -18 | 4 | 62 |
| Left cerebellum | 19 | 3.62 | -8 | -70 | -32 |
| Right primary visual cortex | 41 | 3.56 | 28 | -70 | 12 |
| Right lingual gyrus | 44 | 3.56 | 12 | -54 | -4 |
| Right middle frontal gyrus | 60 | 3.55 | 36 | 42 | 24 |
| Left insula | 10 | 3.47 | -34 | -2 | 14 |
| Left middle occipital cortex | 17 | 3.42 | -32 | -68 | 34 |
| Left postcentral gyrus | 13 | 3.42 | -64 | -14 | 28 |
| Left postcentral gyrus | 8 | 3.35 | -54 | -18 | 30 |
| Right frontal eye fields | 13 | 3.33 | 31 | 20 | 34 |
| Left inferior frontal gyrus, opercular part | 8 | 3.25 | -54 | 16 | 10 |
| Right primary auditory | 6 | 3.25 | 42 | -24 | 2 |
| Right dorsal anterior cingulate cortex | 7 | 3.23 | 8 | 2 | 36 |
| *HC < PTSD+BPD* |  |  |  |  |  |
| No suprathreshold clusters | | | | | |
| *HC > PTSD+BPD+CPD* |  |  |  |  |  |
| Left supramarginal gyrus | 25 | 3.99 | -46 | -32 | 32 |
| Right superior parietal lobe | 22 | 3.93 | 24 | -52 | 36 |
| Right cerebellum | 9 | 3.44 | 28 | -68 | -44 |
| *HC < PTSD+BPD+CPD* |  |  |  |  |  |
| Left middle temporal gyrus | 147 | 4.81 | -66 | -28 | -6 |
| Left middle temporal gyrus | 27 | 3.73 | -32 | 28 | 32 |
| Left rolandic operculum | 14 | 3.71 | -44 | -24 | 20 |
| Right hippocampus | 100 | 3.71 | 24 | -10 | -20 |
| Left inferior frontal gyrus, pars orbitalis | 42 | 3.66 | -46 | 32 | -16 |
| Left parahippocampal gyrus | 43 | 3.57 | -28 | -14 | -24 |
| Right temporal middle gyrus | 16 | 3.53 | 64 | 2 | -16 |
| Left middle cingulum | 21 | 3.40 | -8 | -36 | 36 |
| Right parahippocampal gyrus | 12 | 3.32 | 34 | -26 | -14 |
| Left angular gyrus | 16 | 3.25 | -46 | -62 | 34 |
| Left frontal middle gyrus | 8 | 3.23 | -24 | 26 | 46 |
| Left anterior cingulum | 5 | 3.21 | -4 | 48 | 10 |
| *PTSD+CPD < PTSD+BPD* |  |  |  |  |  |
| Right inferior temporal gyrus | 27 | 3.63 | 56 | -6 | -32 |
| *PTSD+CPD > PTSD+BPD* |  |  |  |  |  |
| No suprathreshold clusters | | | | | |
| *PTSD+CPD > PTSD+BPD+CPD* |  |  |  |  |  |
| No suprathreshold clusters | | | | | |
| *PTSD+CPD < PTSD+BPD+CPD* |  |  |  |  |  |
| Left rolandic operculum | 74 | 4.50 | -44 | -24 | 20 |
| Right middle temporal gyrus | 143 | 4.43 | 64 | 2 | -16 |
| Left middle temporal gyrus | 224 | 4.30 | -66 | -28 | -6 |
| Right hippocampus | 175 | 4.28 | 34 | -14 | -18 |
| Left middle temporal gyrus | 223 | 4.05 | -62 | -4 | -18 |
| Left middle temporal pole | 27 | 3.97 | -46 | 18 | -30 |
| Left frontal middle gyrus | 55 | 3.96 | -30 | 26 | 32 |
| Left superior temporal gyrus | 96 | 3.93 | -46 | -40 | 6 |
| Left superior occipital cortex | 31 | 3.93 | -14 | -96 | 22 |
| Left parahippocampal gyrus | 196 | 3.91 | -30 | -16 | -22 |
| Left inferior frontal gyrus, pars orbitalis | 116 | 3.82 | -46 | 32 | -16 |
| Left inferior frontal gyrus, pars orbitalis | 40 | 3.72 | -22 | 12 | -18 |
| Right middle temporal pole | 10 | 3.52 | 50 | 20 | -26 |
| Left inferior frontal gyrus, triangular part | 27 | 3.51 | 50 | 30 | 2 |
| Right cerebellum | 27 | 3.48 | 16 | -48 | -24 |
| Left insula | 22 | 3.42 | -34 | -2 | -12 |
| Right inferior frontal gyrus, pars orbitalis | 6 | 3.32 | 42 | 40 | -12 |
| Left inferior temporal gyrus | 23 | 3.31 | -46 | -38 | -16 |
| Left angular gyrus | 24 | 3.30 | -46 | -60 | 30 |
| Left superior temporal gyrus | 5 | 3.29 | -68 | -20 | 6 |
| Left middle cingulum | 22 | 3.23 | -6 | -34 | 42 |
| *PTSD+BPD > PTSD+BPD+CPD* |  |  |  |  |  |
| No suprathreshold clusters | | | | | |
| *PTSD+BPD < PTSD+BPD+CPD* |  |  |  |  |  |
| Left rolandic operculum | 330 | 4.85 | -44 | -24 | 18 |
| Left middle frontal gyrus | 64 | 4.00 | -32 | 28 | 30 |
| Left middle cingulum | 75 | 3.87 | -10 | -28 | 36 |
| Right dorsal anterior cingulate cortex | 33 | 3.82 | 12 | 6 | 36 |
| Left superior longitudinal fasciculus | 23 | 3.82 | -32 | -4 | 28 |
| Left primary sensory cortex | 67 | 3.58 | -68 | -14 | 26 |
| Left superior temporal gyrus | 89 | 3.43 | -60 | -2 | 2 |
| Right superior temporal gyrus | 24 | 3.42 | 70 | -20 | 8 |
| Left visual association cortex | 7 | 3.38 | -36 | -64 | 8 |
| Left middle temporal gyrus | 10 | 3.33 | -52 | -36 | -12 |
| Left insula | 17 | 3.33 | -38 | 0 | -6 |
| Left inferior frontal gyrus, opercular part | 19 | 3.31 | -54 | 8 | 24 |
| Right superior temporal gyrus | 25 | 3.23 | 66 | -4 | 2 |
| Left superior temporal gyrus | 11 | 3.21 | -46 | -38 | 8 |
| Left thalamus | 5 | 3.16 | -10 | -16 | 2 |

*Note.* BPD = borderline personality disorder, CPD = cluster c personality disorder, PTSD = posttraumatic stress disorder, HC = healthy controls

**fMRIPrep boilerplate**

Results included in this manuscript come from preprocessing performed using fMRIPrep 21.0.1 (Esteban, Markiewicz, et al. (2018); Esteban, Blair, et al. (2018); RRID:SCR_016216), which is based on Nipype 1.6.1 (K. Gorgolewski et al. (2011); K. J. Gorgolewski et al. (2018); RRID:SCR_002502).

Preprocessing of B0 inhomogeneity mappings

A total of 2 fieldmaps were found available within the input BIDS structure for this particular subject. A B0-nonuniformity map (or fieldmap) was estimated based on two (or more) echo-planar imaging (EPI) references with topup (Andersson, Skare, and Ashburner (2003); FSL 6.0.5.1:57b01774).

Anatomical data preprocessing

A total of 2 T1-weighted (T1w) images were found within the input BIDS dataset. All of them were corrected for intensity non-uniformity (INU) with N4BiasFieldCorrection (Tustison et al. 2010), distributed with ANTs 2.3.3 (Avants et al. 2008, RRID:SCR_004757). The T1w-reference was then skull-stripped with a Nipype implementation of the antsBrainExtraction.sh workflow (from ANTs), using OASIS30ANTs as target template. Brain tissue segmentation of cerebrospinal fluid (CSF), white-matter (WM) and gray-matter (GM) was performed on the brain-extracted T1w using fast (FSL 6.0.5.1:57b01774, RRID:SCR_002823, Zhang, Brady, and Smith 2001). A T1w-reference map was computed after registration of 2 T1w images (after INU-correction) using mri_robust_template (FreeSurfer 6.0.1, Reuter, Rosas, and Fischl 2010). Brain surfaces were reconstructed using recon-all (FreeSurfer 6.0.1, RRID:SCR_001847, Dale, Fischl, and Sereno 1999), and the brain mask estimated previously was refined with a custom variation of the method to reconcile ANTs-derived and FreeSurfer-derived segmentations of the cortical gray-matter of Mindboggle (RRID:SCR_002438, Klein et al. 2017). Volume-based spatial normalization to two standard spaces (MNI152NLin6Asym, MNI152NLin2009cAsym) was performed through nonlinear registration with antsRegistration (ANTs 2.3.3), using brain-extracted versions of both T1w reference and the T1w template. The following templates were selected for spatial normalization: FSL’s MNI ICBM 152 non-linear 6th Generation Asymmetric Average Brain Stereotaxic Registration Model [Evans et al. (2012), RRID:SCR_002823; TemplateFlow ID: MNI152NLin6Asym], ICBM 152 Nonlinear Asymmetrical template version 2009c [Fonov et al. (2009), RRID:SCR_008796; TemplateFlow ID: MNI152NLin2009cAsym].

Functional data preprocessing

For each of the 4 BOLD runs found per subject (across all tasks and sessions), the following preprocessing was performed. First, a reference volume and its skull-stripped version were generated using a custom methodology of fMRIPrep. Head-motion parameters with respect to the BOLD reference (transformation matrices, and six corresponding rotation and translation parameters) are estimated before any spatiotemporal filtering using mcflirt (FSL 6.0.5.1:57b01774, Jenkinson et al. 2002). The estimated fieldmap was then aligned with rigid-registration to the target EPI (echo-planar imaging) reference run. The field coefficients were mapped on to the reference EPI using the transform. BOLD runs were slice-time corrected to 1.07s (0.5 of slice acquisition range 0s-2.15s) using 3dTshift from AFNI (Cox and Hyde 1997, RRID:SCR_005927). The BOLD reference was then co-registered to the T1w reference using bbregister (FreeSurfer) which implements boundary-based registration (Greve and Fischl 2009). Co-registration was configured with six degrees of freedom. Several confounding time-series were calculated based on the preprocessed BOLD: framewise displacement (FD), DVARS and three region-wise global signals. FD was computed using two formulations following Power (absolute sum of relative motions, Power et al. (2014)) and Jenkinson (relative root mean square displacement between affines, Jenkinson et al. (2002)). FD and DVARS are calculated for each functional run, both using their implementations in Nipype (following the definitions by Power et al. 2014). The three global signals are extracted within the CSF, the WM, and the whole-brain masks. Additionally, a set of physiological regressors were extracted to allow for component-based noise correction (CompCor, Behzadi et al. 2007). Principal components are estimated after high-pass filtering the preprocessed BOLD time-series (using a discrete cosine filter with 128s cut-off) for the two CompCor variants: temporal (tCompCor) and anatomical (aCompCor). tCompCor components are then calculated from the top 2% variable voxels within the brain mask. For aCompCor, three probabilistic masks (CSF, WM and combined CSF+WM) are generated in anatomical space. The implementation differs from that of Behzadi et al. in that instead of eroding the masks by 2 pixels on BOLD space, the aCompCor masks are subtracted a mask of pixels that likely contain a volume fraction of GM. This mask is obtained by dilating a GM mask extracted from the FreeSurfer’s aseg segmentation, and it ensures components are not extracted from voxels containing a minimal fraction of GM. Finally, these masks are resampled into BOLD space and binarized by thresholding at 0.99 (as in the original implementation). Components are also calculated separately within the WM and CSF masks. For each CompCor decomposition, the k components with the largest singular values are retained, such that the retained components’ time series are sufficient to explain 50 percent of variance across the nuisance mask (CSF, WM, combined, or temporal). The remaining components are dropped from consideration. The head-motion estimates calculated in the correction step were also placed within the corresponding confounds file. The confound time series derived from head motion estimates and global signals were expanded with the inclusion of temporal derivatives and quadratic terms for each (Satterthwaite et al. 2013). Frames that exceeded a threshold of 0.5 mm FD or 1.5 standardised DVARS were annotated as motion outliers. The BOLD time-series were resampled into standard space, generating a preprocessed BOLD run in MNI152NLin6Asym space. First, a reference volume and its skull-stripped version were generated using a custom methodology of fMRIPrep. The BOLD time-series were resampled onto the following surfaces (FreeSurfer reconstruction nomenclature): fsnative, fsaverage5. Automatic removal of motion artifacts using independent component analysis (ICA-AROMA, Pruim et al. 2015) was performed on the preprocessed BOLD on MNI space time-series after removal of non-steady state volumes and spatial smoothing with an isotropic, Gaussian kernel of 6mm FWHM (full-width half-maximum). Corresponding “non-aggresively” denoised runs were produced after such smoothing. Additionally, the “aggressive” noise-regressors were collected and placed in the corresponding confounds file. All resamplings can be performed with a single interpolation step by composing all the pertinent transformations (i.e. head-motion transform matrices, susceptibility distortion correction when available, and co-registrations to anatomical and output spaces). Gridded (volumetric) resamplings were performed using antsApplyTransforms (ANTs), configured with Lanczos interpolation to minimize the smoothing effects of other kernels (Lanczos 1964). Non-gridded (surface) resamplings were performed using mri_vol2surf (FreeSurfer).

Many internal operations of fMRIPrep use Nilearn 0.8.1 (Abraham et al. 2014, RRID:SCR_001362), mostly within the functional processing workflow. For more details of the pipeline, see the section corresponding to workflows in fMRIPrep’s documentation.

Copyright Waiver

The above boilerplate text was automatically generated by fMRIPrep with the express intention that users should copy and paste this text into their manuscripts unchanged. It is released under the CC0 license.

References

Abraham, Alexandre, Fabian Pedregosa, Michael Eickenberg, Philippe Gervais, Andreas Mueller, Jean Kossaifi, Alexandre Gramfort, Bertrand Thirion, and Gael Varoquaux. 2014. “Machine Learning for Neuroimaging with Scikit-Learn.” Frontiers in Neuroinformatics 8. https://doi.org/10.3389/fninf.2014.00014.

Andersson, Jesper L. R., Stefan Skare, and John Ashburner. 2003. “How to Correct Susceptibility Distortions in Spin-Echo Echo-Planar Images: Application to Diffusion Tensor Imaging.” NeuroImage 20 (2): 870–88. https://doi.org/10.1016/S1053-8119(03)00336-7.

Avants, B. B., C. L. Epstein, M. Grossman, and J. C. Gee. 2008. “Symmetric Diffeomorphic Image Registration with Cross-Correlation: Evaluating Automated Labeling of Elderly and Neurodegenerative Brain.” Medical Image Analysis 12 (1): 26–41. https://doi.org/10.1016/j.media.2007.06.004.

Behzadi, Yashar, Khaled Restom, Joy Liau, and Thomas T. Liu. 2007. “A Component Based Noise Correction Method (CompCor) for BOLD and Perfusion Based fMRI.” NeuroImage 37 (1): 90–101. https://doi.org/10.1016/j.neuroimage.2007.04.042.

Cox, Robert W., and James S. Hyde. 1997. “Software Tools for Analysis and Visualization of fMRI Data.” NMR in Biomedicine 10 (4-5): 171–78. https://doi.org/10.1002/(SICI)1099-1492(199706/08)10:4/5<171::AID-NBM453>3.0.CO;2-L.

Dale, Anders M., Bruce Fischl, and Martin I. Sereno. 1999. “Cortical Surface-Based Analysis: I. Segmentation and Surface Reconstruction.” NeuroImage 9 (2): 179–94. https://doi.org/10.1006/nimg.1998.0395.

Esteban, Oscar, Ross Blair, Christopher J. Markiewicz, Shoshana L. Berleant, Craig Moodie, Feilong Ma, Ayse Ilkay Isik, et al. 2018. “fMRIPrep.” Software. https://doi.org/10.5281/zenodo.852659.

Esteban, Oscar, Christopher Markiewicz, Ross W Blair, Craig Moodie, Ayse Ilkay Isik, Asier Erramuzpe Aliaga, James Kent, et al. 2018. “fMRIPrep: A Robust Preprocessing Pipeline for Functional MRI.” Nature Methods. https://doi.org/10.1038/s41592-018-0235-4.

Evans, AC, AL Janke, DL Collins, and S Baillet. 2012. “Brain Templates and Atlases.” NeuroImage 62 (2): 911–22. https://doi.org/10.1016/j.neuroimage.2012.01.024.

Fonov, VS, AC Evans, RC McKinstry, CR Almli, and DL Collins. 2009. “Unbiased Nonlinear Average Age-Appropriate Brain Templates from Birth to Adulthood.” NeuroImage 47, Supplement 1: S102. https://doi.org/10.1016/S1053-8119(09)70884-5.

Gorgolewski, K., C. D. Burns, C. Madison, D. Clark, Y. O. Halchenko, M. L. Waskom, and S. Ghosh. 2011. “Nipype: A Flexible, Lightweight and Extensible Neuroimaging Data Processing Framework in Python.” Frontiers in Neuroinformatics 5: 13. https://doi.org/10.3389/fninf.2011.00013.

Gorgolewski, Krzysztof J., Oscar Esteban, Christopher J. Markiewicz, Erik Ziegler, David Gage Ellis, Michael Philipp Notter, Dorota Jarecka, et al. 2018. “Nipype.” Software. https://doi.org/10.5281/zenodo.596855.

Greve, Douglas N, and Bruce Fischl. 2009. “Accurate and Robust Brain Image Alignment Using Boundary-Based Registration.” NeuroImage 48 (1): 63–72. https://doi.org/10.1016/j.neuroimage.2009.06.060.

Jenkinson, Mark, Peter Bannister, Michael Brady, and Stephen Smith. 2002. “Improved Optimization for the Robust and Accurate Linear Registration and Motion Correction of Brain Images.” NeuroImage 17 (2): 825–41. https://doi.org/10.1006/nimg.2002.1132.

Klein, Arno, Satrajit S. Ghosh, Forrest S. Bao, Joachim Giard, Yrjö Häme, Eliezer Stavsky, Noah Lee, et al. 2017. “Mindboggling Morphometry of Human Brains.” PLOS Computational Biology 13 (2): e1005350. https://doi.org/10.1371/journal.pcbi.1005350.

Lanczos, C. 1964. “Evaluation of Noisy Data.” Journal of the Society for Industrial and Applied Mathematics Series B Numerical Analysis 1 (1): 76–85. https://doi.org/10.1137/0701007.

Power, Jonathan D., Anish Mitra, Timothy O. Laumann, Abraham Z. Snyder, Bradley L. Schlaggar, and Steven E. Petersen. 2014. “Methods to Detect, Characterize, and Remove Motion Artifact in Resting State fMRI.” NeuroImage 84 (Supplement C): 320–41. https://doi.org/10.1016/j.neuroimage.2013.08.048.

Pruim, Raimon H. R., Maarten Mennes, Daan van Rooij, Alberto Llera, Jan K. Buitelaar, and Christian F. Beckmann. 2015. “ICA-AROMA: A Robust ICA-Based Strategy for Removing Motion Artifacts from fMRI Data.” NeuroImage 112 (Supplement C): 267–77. https://doi.org/10.1016/j.neuroimage.2015.02.064.

Reuter, Martin, Herminia Diana Rosas, and Bruce Fischl. 2010. “Highly Accurate Inverse Consistent Registration: A Robust Approach.” NeuroImage 53 (4): 1181–96. https://doi.org/10.1016/j.neuroimage.2010.07.020.

Satterthwaite, Theodore D., Mark A. Elliott, Raphael T. Gerraty, Kosha Ruparel, James Loughead, Monica E. Calkins, Simon B. Eickhoff, et al. 2013. “An improved framework for confound regression and filtering for control of motion artifact in the preprocessing of resting-state functional connectivity data.” NeuroImage 64 (1): 240–56. https://doi.org/10.1016/j.neuroimage.2012.08.052.

Tustison, N. J., B. B. Avants, P. A. Cook, Y. Zheng, A. Egan, P. A. Yushkevich, and J. C. Gee. 2010. “N4itk: Improved N3 Bias Correction.” IEEE Transactions on Medical Imaging 29 (6): 1310–20. https://doi.org/10.1109/TMI.2010.2046908.

Zhang, Y., M. Brady, and S. Smith. 2001. “Segmentation of Brain MR Images Through a Hidden Markov Random Field Model and the Expectation-Maximization Algorithm.” IEEE Transactions on Medical Imaging 20 (1): 45–57. https://doi.org/10.1109/42.906424.
